# Idiopathic Pneumonia Syndrome and Pulmonary Toxicity After Total Body Irradiation for Bone Marrow Transplantation in the Modern Transplant Era

**DOI:** 10.3390/curroncol33090545

**Published:** 2026-09-09

**Authors:** Ruijia Jin, Justin Oh, Iman Baharmand, Matthew Chan, Jessica Chan, Andrea C. Lo, Cassidy Northway, Conrad Yuen, I. A. Popescu, T. P. L. Nghiem, Yasser Abou Mourad, Cheryl Duzenli

**Affiliations:** 1 Department of Radiation Oncology, BC Cancer, Vancouver, BC V5Z 4E6, Canada; rachel.jin@bccancer.bc.ca (R.J.); justin.oh@bccancer.bc.ca (J.O.); matthew.chan@bccancer.bc.ca (M.C.); jessica.chan@bccancer.bc.ca (J.C.); andrea.lo@bccancer.bc.ca (A.C.L.); 2 Department of Family Practice, University of British Columbia, Vancouver, BC V6T 1Z3, Canada; 3 Department of Physics and Astronomy, University of British Columbia, Vancouver, BC V6T 1Z1, Canada; cassidy.northway@bccancer.bc.ca (C.N.); tpopescu@bccancer.bc.ca (I.A.P.); 4 Department of Medical Physics, BC Cancer, Vancouver, BC V5Z 4E6, Canada; cyuen@bccancer.bc.ca; 5 Department of Clinical Research, BC Cancer, Vancouver, BC V5Z 4E6, Canada; thi.nghiem@bccancer.bc.ca; 6 Leukemia/BMT Program of BC, Vancouver General Hospital, Vancouver, BC V5Z 1M9, Canada; ymourad@bccancer.bc.ca; 7 Division of Hematology, Faculty of Medicine, University of British Columbia, Vancouver, BC V5Z 1M9, Canada

**Keywords:** acute graft vs. host disease, acute lymphoid leukemia, acute myeloid leukemia, allogeneic hematopoietic stem cell transplantation, chronic graft vs. host disease, idiopathic pneumonia syndrome, myelodysplastic syndrome (MDS), pulmonary infection, pulmonary toxicity, total body irradiation

## Abstract

Total body irradiation (TBI) is commonly used for myeloablative conditioning prior to allogeneic hematopoietic stem cell transplantation (HSCT) in high-risk, relapsed, or refractory hematologic malignancies. Pulmonary toxicity (PT)—including pneumonia, diffuse alveolar hemorrhage, and interstitial pneumonitis—is a serious complication after HSCT. Idiopathic pneumonia syndrome (IPS), defined as widespread alveolar injury without cardiogenic or infectious causes, represents a non-infectious form of PT. This study characterizes PT and IPS and explores associated factors following TBI-based HSCT in adults. We conducted a retrospective review of a prospectively collected database of adult patients treated with TBI-based HSCT from 2015 to 2022. Clinical records and chest imaging were reviewed to identify PT and IPS. IPS occurred in 20 patients (18%) of the cohort, with 8 patients (7%) within 100 days and 13 patients (13%) after 100 days. One patient experienced both early- and late-onset IPS and was counted once in the cumulative analysis. Early pulmonary infection was associated with reduced overall survival in both univariable (*p* = 0.002) and multivariable 100-day landmark analyses (*p* = 0.047). Acute graft-versus-host disease (GVHD) was independently associated with improved survival (*p* = 0.017), whereas chronic GVHD was not statistically significant in the landmark analyses. PT and IPS incidences were consistent with previously published adult TBI cohorts. While no significant clinical or radiation-related predictors of IPS were identified, pulmonary infection emerged as a predictor of inferior overall survival.

## Simple Summary

Total body irradiation (TBI) is commonly used before bone marrow transplantation to treat blood cancers. Although effective, TBI can lead to pulmonary toxicity and a rare condition called idiopathic pneumonia syndrome (IPS). In this population-based study, we analyzed outcomes in all adult patients in British Columbia who received myeloablative TBI prior to allogeneic hematopoietic stem cell transplantation. We examined the incidence and outcomes of pulmonary toxicity and IPS and explored associated clinical and radiation-related factors. IPS incidence was low and consistent with previously published adult TBI cohorts using standardized ATS diagnostic criteria. Pulmonary infections were more common and more strongly associated with reduced survival. Overall, this study represents a contemporary benchmark for IPS in the modern transplant era. Further prospective multi-center studies with large numbers of patients are warranted for analyses.

## 1. Introduction

Total body irradiation (TBI) is an established component of myeloablative conditioning regimens for allogeneic hematopoietic stem cell transplantation (HSCT). Recent randomized and multicenter studies have demonstrated improved disease control, lower relapse rates, and improved overall survival with TBI-based conditioning compared with non-TBI-based regimens in selected pediatric and adult patients [1,2].

Despite these benefits, TBI may be associated with significant pulmonary complications, including pulmonary toxicity (PT) and idiopathic pneumonia syndrome (IPS), which remain important causes of post-transplant morbidity and mortality [3]. Reported incidences of PT and IPS vary widely (0–71% and 1–60%, respectively), reflecting differences in conditioning intensity, lung shielding techniques, radiation dose rates, and, importantly, inconsistent diagnostic definitions across studies [3]. The distinction between infectious pulmonary complications and IPS remains particularly challenging because IPS is a diagnosis of exclusion. The American Thoracic Society (ATS) defines IPS as widespread alveolar injury occurring after HSCT in the absence of an identifiable infectious, cardiac, renal, or volume overload etiology [4].

Several clinical and treatment-related factors have been associated with pulmonary complications following HSCT. TBI-based conditioning, higher lung radiation dose, increased dose rate, acute graft-versus-host disease (GVHD), and donor source have all been implicated in previous studies, although findings have been inconsistent [5,6,7]. More recently, intensity-modulated TBI techniques, such as volumetric modulated arc therapy (VMAT), have demonstrated improved lung dose sparing; however, the optimal lung dose and dose rate for minimizing pulmonary toxicity while maintaining transplant efficacy remain uncertain [8,9,10].

Given the variability in reported IPS incidence and the lack of standardized contemporary data, the primary aim of this study was to determine the incidence of PT and IPS using standardized ATS diagnostic criteria in adults receiving TBI-based myeloablative conditioning and to evaluate their association with overall survival. Secondary objectives were to explore associated clinical and radiation-related factors.

## 2. Materials and Methods

### 2.1. Study Design

We performed a retrospective review of a prospectively collected database to analyze clinical outcomes and radiation metrics in all adult patients treated with TBI for myeloablative conditioning prior to HSCT in British Columbia from 2015 to 2022. We selected 2015 as the starting year because the mid-2010s marked transformative advances in GVHD prophylaxis and supportive care strategies. This allows our cohort to be analyzed in the modern transplant era [11,12]. This resulted in a median 4-year follow-up horizon, allowing for assessment of both acute and chronic pulmonary sequelae. Most late pulmonary complications tend to manifest within the first 2–5 years post-transplant [6].

### 2.2. Patient Selection

Patients included in this study were ≥18 years old at the time of diagnosis and had malignant disease that was treated with TBI myeloablative conditioning before allogeneic HSCT from 2015 to 2022. Exclusion criteria included patients who received reduced-intensity conditioning, patients who received bone marrow transplantation for benign conditions, and pediatric patients (age < 18). This study was approved by the Institutional Research Ethics Board of BC Cancer.

### 2.3. Radiation Technique

TBI was delivered using Cobalt-60 with a sweeping beam technique. Patients were treated with two fields: one in the supine position and one in the prone position [13]. Patients prescribed TBI whole body dose of 1200 cGy in six fractions or 1350 cGy in nine fractions were included. A lung compensator was used to ensure the lungs received the prescription dose, without exceeding it. Treatment time in this study refers to the time to deliver one field, for reporting of dose rate. Variation in treatment time is a function of both patient size and Cobalt-60 source strength. During the period of the study, the Cobalt source was replaced once it reached its half-life, contributing to a systematic 50% variation in dose rate over the course of the study.

### 2.4. Radiation Dose Simulation

Treatment planning was performed on CT simulation images acquired in the prone and supine positions. Since the technique was designed to provide a uniform dose to the whole body, including the lungs, the inter- and intra-patient variation in lung dose was expected to be low. Full body dose distributions were simulated using Monte Carlo methods [14] for a representative patient sample including 5 male and 5 female patients ranging in age from 21 to 52 years, having received a prescribed TBI dose of 1200 cGy. Patients in the range of height and weight spanning the study population were chosen without prior knowledge of the clinical outcome data. Mean lung dose and V100% lung were captured for this sample. Mid-separation lung point dose rate was reported for all patients.

### 2.5. Data Abstraction

All patient data were anonymized using a unique study code. Abstracted data from electronic medical records included patient clinical characteristics, including age at diagnosis, disease requiring TBI/HSCT, pre-HSCT lung disease, tobacco use, and Eastern Cooperative Oncology Group (ECOG) performance status at baseline; treatment-related characteristics such as conditioning regimen, donor source, GVHD prophylaxis, and TBI parameters including dose, mid-lung dose rates, treatment time, and fractionation pattern. In addition, researchers recorded primary versus recurrent disease, presence of extramedullary disease, and minimal residual disease (MRD) status prior to transplant. Baseline pulmonary function test (PFT) results, pre-HSCT chest X-ray (CXR), pre-HSCT bronchoscopy findings and CT chest findings were reviewed when available. Post-treatment investigations, including post-HSCT PFT results, bronchoscopy, CXR, and CT chest results, were reviewed and compared to pre-HSCT investigations of the same type. Bronchoscopy with bronchoalveolar lavage (BAL) was performed when clinically indicated, and microbiological investigations identified a range of bacterial, fungal, and mycobacterial pathogens in selected patients. Post-treatment outcome data collected included date of last follow-up, relapse, type of relapse, graft-versus-host disease, complications, and death. The available investigations were reviewed by a radiation oncologist or radiation oncology resident.

### 2.6. Definition of PT, IPS, and PI

Based on criteria commonly used in previous studies [3,15], PT following TBI-based HSCT was defined by meeting at least one of the following criteria: (1) the presence of at least two out of three clinical symptoms—dyspnea or other respiratory complaints, fever, and hypoxia or cyanosis; (2) radiologic findings, including bilateral diffuse opacities or increased interstitial markings on chest radiograph, or diffuse ground glass opacities on chest CT; or (3) abnormal pulmonary function tests showing either restrictive or obstructive ventilatory defects.

Idiopathic pneumonia syndrome (IPS) was defined according to standardized American Thoracic Society (ATS) criteria as an idiopathic syndrome of pneumopathy occurring after HSCT [3,4]. IPS was defined as a diagnosis of exclusion where diagnostic evaluation was based on review of the clinical record and typically included chest imaging together with microbiological investigations performed only as clinically indicated, including bronchoalveolar lavage, sputum cultures, blood cultures, and viral testing where available. Diagnosis required the presence of one or more clinical symptoms (dyspnea, cough, +/− hypoxemia), in conjunction with new multilobar (typically diffuse or patchy bilateral) infiltrates on chest imaging. In addition, there must be no cardiogenic pulmonary edema or renal failure/fluid overload explaining the patient’s clinical or radiologic findings.

Pulmonary infection (PI) was defined as a subset of pulmonary toxicity with an identifiable infectious etiology. These included cases confirmed by positive microbiologic testing or those assessed by clinician documentation, which may include radiographic findings consistent with pneumonia, accompanied by clinical features such as fever, elevated inflammatory markers, and/or response to antimicrobial therapy [4].

### 2.7. Statistical Analysis

The primary objective was to characterize the contemporary incidence of pulmonary toxicity (PT) and idiopathic pneumonia syndrome (IPS) following TBI-based myeloablative conditioning and to evaluate their association with overall survival. A secondary objective was to explore clinical and radiation-related factors associated with IPS. Comparisons of baseline characteristics between patients with and without IPS were performed using the Wilcoxon rank-sum test for continuous variables and Fisher’s exact test for categorical variables, as appropriate.

As a secondary outcome, overall survival (OS) was evaluated using Cox proportional hazards regression. Univariable Cox analyses were first performed, and variables with *p*-values < 0.20 were entered into multivariable Cox models. Model selection was performed using a stepwise approach based on the Akaike Information Criterion (AIC) to identify independent predictors of OS. Hazard ratios (HRs) with 95% confidence intervals (CIs) were reported.

Because the exact timing of IPS onset was unavailable for all patients, IPS could not be modeled as a time-dependent covariate. To minimize immortal time bias, prespecified landmark analyses were performed using a 100-day landmark. Patients alive at day 100 after HSCT were stratified according to the presence or absence of IPS or pulmonary toxicity occurring within the first 100 days, and overall survival was estimated from the landmark timepoint using the Kaplan–Meier method and compared using the log-rank test. Landmark univariable and multivariable Cox regression analyses were subsequently performed to identify factors associated with overall survival. The proportional hazards assumption was assessed for the final multivariable models and was satisfied.

All statistical analyses were performed using R version 4.3.1 (R: A language and environment for statistical computing. R Foundation for Statistical Computing, Vienna, Austria. URL https://www.R-project.org/). *p*-values < 0.05 were considered statistically significant.

## 3. Results

### 3.1. Patient Characteristics and Radiation Treatment

Demographics and clinical characteristics for the 110 patients who received TBI-based myeloablative conditioning prior to allogeneic HSCT are summarized in Table 1.

The median age at cancer diagnosis was 35.9 years (IQR 26.1–44.8). Acute lymphoblastic leukemia (ALL) was the most common underlying malignancy (50/110, 45.5%), followed by acute myeloid leukemia/myelodysplastic syndrome (AML/MDS) (24/110, 21.8%), non-Hodgkin lymphoma (NHL) (19/110, 17.3%), and others (17/110, 15.4%). Most patients had ECOG performance status < 2 at baseline (107/110, 97.3%), and 22 patients (20.0%) had pre-existing respiratory comorbidities. Tobacco use was reported in 23/110 (20.9%) of the cohort. The majority of patients (98/110, 89%) received a TBI dose of 1200 cGy in six fractions; the remaining patients (12/110, 11%) received 1350 cGy in nine fractions. Median mid-lung dose rate was 11.1 cGy/min (IQR 8.26–13.37). Using Monte Carlo simulation of a representative patient sample, the median mean lung dose was 1090 cGy (Range 1050–1140 cGy) providing an estimate of mean lung dose for the population of 91% ± 5% of the prescribed dose. Median V100% (Volume of lung receiving ≥ 100% of the prescribed dose) was 5% with a range of 0–15% of the lung volume. The dose–volume histogram data are available in Supplementary Materials.

### 3.2. Pulmonary Toxicity and Idiopathic Pneumonia Syndrome

Eight patients (7.3%) developed early-onset (within 100 days) IPS defined per ATS criteria. 13 patients (13.3%) were diagnosed with late-onset (after 100 days) IPS, including one patient who had previously developed early IPS; therefore, 20 unique patients experienced IPS. A total of 51 patients (46.4%) developed PT within 100 days post-transplant, while 79 patients cumulatively developed PT (71.8%). Detailed comparisons between patients with and without PT are shown in Table 2 and Table 3. None of the evaluated clinical characteristics or radiation parameters, including age, ECOG performance status, HLA match status, respiratory comorbidity, tobacco use, prescribed dose, treatment time, TBI mid-lung dose rate, or GVHD, were significantly associated with PT.

Within 100 days following HSCT, pulmonary infection occurred in 72 patients (65.5%), while bloodstream infections occurred in 29 patients (26.4%). Pulmonary infection was diagnosed using clinical, radiographic, and microbiological data obtained during routine clinical care. Microbiological confirmation was based on bronchoalveolar lavage (BAL), sputum cultures, blood cultures, fungal biomarkers, viral testing, or other clinically indicated investigations where available. In the absence of microbiological confirmation, pulmonary infection was diagnosed when multidisciplinary clinical assessment concluded that the pulmonary findings were most consistent with an infectious etiology.

Post-transplant bronchoscopy was performed in 24 patients. Eleven bronchoscopies occurred within 100 days of HSCT and 13 occurred thereafter. Within the first 100 days, clinically significant pathogens were identified in two patients (one acid-fast bacillary/mycobacterial organism and one Strongyloides infection), while two bronchoscopies demonstrated normal respiratory flora only and seven demonstrated no identifiable pathogen. Beyond 100 days, five clinically significant pathogens were identified, including three fungal infections (one Aspergillus fumigatus and two invasive pulmonary aspergillosis cases with positive galactomannan), one bacterial infection (Moraxella spp.), and one viral infection (adenovirus).

### 3.3. IPS and Clinical Predictors

Detailed comparisons between patients with and without IPS are provided in Table 4 and Table 5. Patients with IPS within 100 days were older (median age: 43.0 vs. 35.0 years; *p* = 0.0699) and more likely to have an ECOG performance status > 0 (87.5% vs. 61.8%; *p* = 0.254), though differences did not reach statistical significance. There is no significant difference in Human Leukocyte Antigen (HLA) match status, respiratory comorbidities, tobacco use, or TBI dose parameters for patients with and without IPS within 100 days.

In an extended analysis, including all IPS cases (n = 20), patients with IPS tended to be older (median 39.7 vs. 34.0 years, *p* = 0.074) with lower rates of tobacco use (5.0% vs. 24.4%, *p* = 0.0682), though again not reaching statistical significance. Radiation treatment characteristics, including dose, treatment time, and midplane dose rates, were not significantly associated with IPS development.

**Figure 1:**
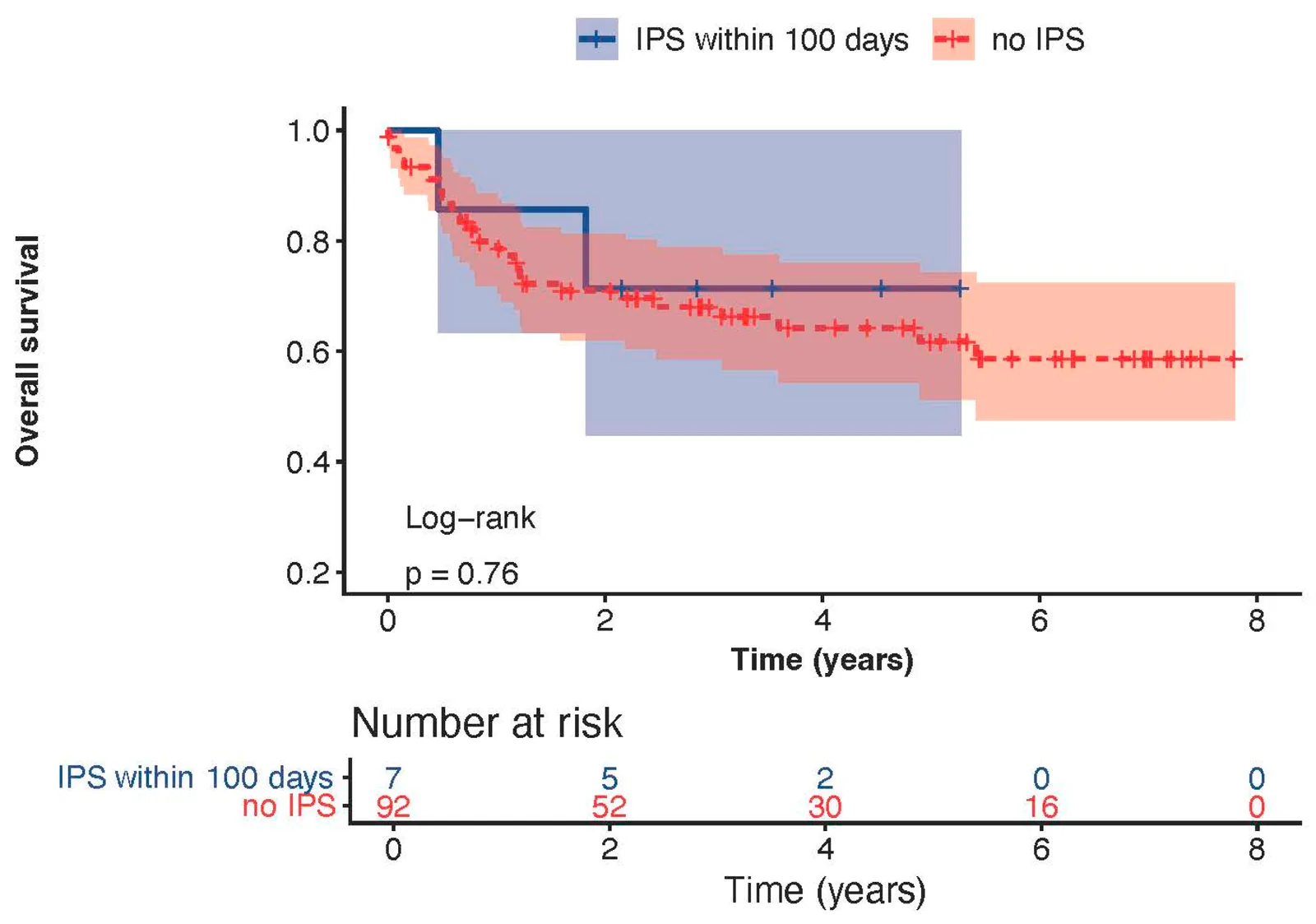
100-day landmark KM curve: IPS within 100 days.

**Figure 2:**
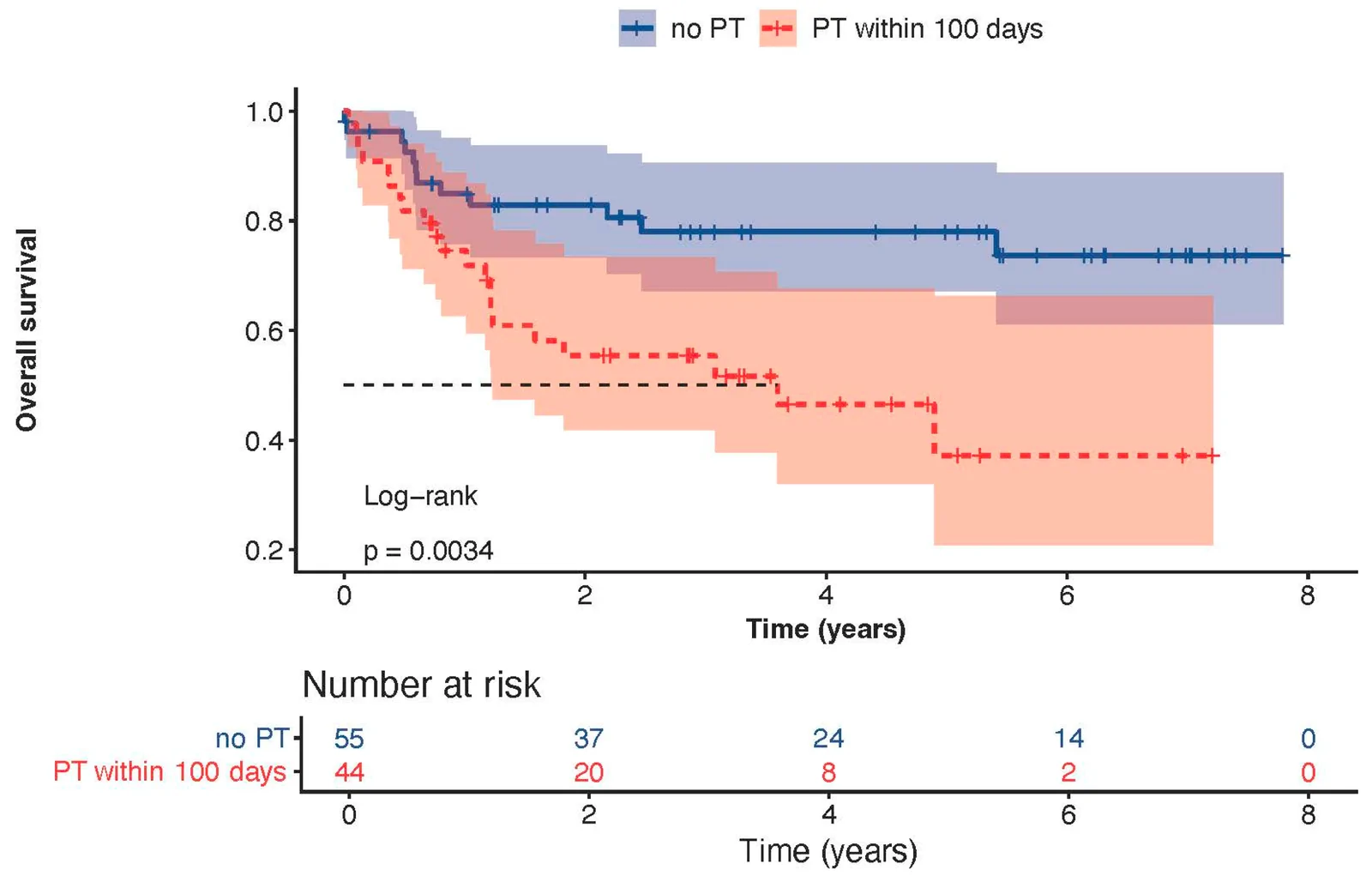
100-day landmark KM curve: PT within 100 days.

To account for immortal time bias, landmark analyses were performed using a 100-day landmark (Figure 1 and Figure 2). Kaplan–Meier analyses demonstrated no significant difference in overall survival according to IPS within the first 100 days (*p* = 0.76). Pulmonary toxicity within the first 100 days was, however, associated with worse overall survival (*p* = 0.0034).

### 3.4. Clinical Outcomes and Pulmonary Function

Pulmonary function tests (PFTs) were available for only a limited subset of patients and these analyses should therefore be considered exploratory. For the 26 patients with both pre-BMT PFTs and post-BMT PFTs available, the median change in FEV1 was −0.15 L and median change in DLCO was −4.0%. In those without PT (13 patients), median FEV1 change was −0.12 L and median DLCO change was −2%; in those with PT (13 patients), median FEV1 change was −0.19 L and median DLCO change was −9%. This shows a trend towards a greater decline in DLCO and FEV1 post-transplant among those with PT, consistent with restrictive and diffusion impairment patterns, though formal statistical comparisons were limited due to sample size.

### 3.5. Survival and Prognostic Factors

To account for potential immortal time bias, a prespecified 100-day landmark analysis was performed. In the landmark univariable analysis, (Table 6), pulmonary infection within the first 100 days was significantly associated with inferior overall survival (HR 3.02, 95% CI 1.50–6.09, *p* = 0.002), whereas IPS was unassociated with survival (HR 1.25, 95% CI 0.30–5.25, *p* = 0.80). Acute GVHD was associated with improved overall survival (HR 0.46, 95% CI 0.23–0.92, *p* = 0.027), whereas chronic GVHD was not significantly associated with survival (HR 0.59, 95% CI 0.29–1.18, *p* = 0.13).

A multivariable Cox model (Table 7), including variables with univariate *p* < 0.20, identified disease relapse (HR 3.24, 95% CI 1.35–7.74, *p* = 0.008), acute GVHD (HR 0.38, 95% CI 0.17–0.85, *p* = 0.017), and pulmonary infection within 100 days (HR 2.38, 95% CI 1.01–5.62, *p* = 0.047) as independent predictors of overall survival. Salvage treatment intent demonstrated a trend toward inferior survival (HR 2.70, 95% CI 0.87–8.35, *p* = 0.085).

The final stepwise-selected model (Table 8) confirmed salvage treatment intent (HR 2.92, 95% CI 1.09–7.84, *p* = 0.034), disease relapse (HR 3.69, 95% CI 1.58–8.62, *p* = 0.003), pulmonary infection within 100 days (HR 2.57, 95% CI 1.13–5.84, *p* = 0.024), and acute GVHD (HR 0.44, 95% CI 0.21–0.95, *p* = 0.036) as independent predictors of overall survival. Although CNS involvement was retained in the final model, it was not statistically significant (HR 2.72, 95% CI 0.76–9.74, *p* = 0.12).

## 4. Discussion

Several high-quality studies have demonstrated that total body irradiation (TBI) remains an effective component of myeloablative conditioning prior to allogeneic HSCT, improving disease control and reducing relapse in selected hematologic malignancies [1,2,16]. However, pulmonary complications remain an important cause of post-transplant morbidity and mortality [17,18,19]. In this population-based cohort of adults treated with TBI-based myeloablative conditioning, we found that idiopathic pneumonia syndrome (IPS) was uncommon, whereas pulmonary infection occurred substantially more frequently and was independently associated with inferior overall survival. The incidence of PT within 100 days was 46% and cumulatively 72%. These findings provide contemporary benchmark data for pulmonary complications following conventional TBI in the modern transplant era.

A major challenge in studying IPS is the lack of consistency in its definition across previous studies. As highlighted by Vogel et al., reported IPS incidence ranges from 1% to 60%, reflecting heterogeneous diagnostic criteria and inconsistent exclusion of infectious etiologies [3]. IPS remains a diagnosis of exclusion, requiring compatible clinical and radiographic findings together with exclusion of infectious, cardiac, renal, and volume overload causes [7]. In retrospective studies, including ours, the extent of microbiological evaluation is inevitably influenced by clinical practice, and bronchoscopy or bronchoalveolar lavage (BAL) is not performed routinely in all patients [20,21]. Consequently, variation in diagnostic workup may contribute to differences in reported IPS incidence between studies.

Within this relatively homogeneous cohort treated using a standardized Cobalt-60 sweeping-beam TBI technique with lung compensation, no significant associations were identified between PT or IPS and the clinical or radiation-related variables evaluated, including prescribed TBI dose, treatment time, or dose rate. By design, all patients received a relatively homogeneous lung dose and while this study cannot exclude a relationship between lung dose or dose rate and IPS risk, our results suggest that within the range of radiation parameters evaluated, no statistically significant associations were identified.

Using standardized ATS diagnostic criteria, we observed an early IPS incidence of 7.3%, which is consistent with previously published adult TBI cohorts [3,6,22]. IPS was not associated with inferior overall survival in our 100-day landmark analyses. However, as in previous studies [3,22], the relatively small number of IPS events (n = 20 overall; n = 8 within 100 days) limited our ability to detect meaningful associations. Prospective multicenter studies including larger patient cohorts are needed to draw more definitive conclusions and develop clinically useful risk prediction models.

In our study, pulmonary infection occurring within the first 100 days remained independently associated with inferior overall survival in both univariable and multivariable landmark analyses. While these findings match previous reports that pulmonary infections are more common than IPS and are more strongly associated with reduced survival [6,17,18], this study confirms that this remains the case in recent years despite advances in transplant practice over the past decade, including improvements in antimicrobial prophylaxis, microbiological diagnostics, GVHD management, and supportive care.

An important limitation of this study is the retrospective assessment of IPS and pulmonary infection. Although standardized ATS criteria were applied, microbiological investigations were not performed according to a predefined protocol. Bronchoscopy with BAL was undertaken only when clinically indicated, reflecting routine clinical practice. Consequently, some degree of misclassification between infectious pulmonary complications and IPS cannot be excluded. In addition, we were unable to distinguish dysbiosis from clinically significant infection or evaluate changes in microbiological findings following antimicrobial therapy. This limitation should be considered when interpreting comparisons between IPS and pulmonary infection. In addition, the observed association between pulmonary infection and overall survival should be interpreted as an association with clinically ascertained pulmonary infection rather than exclusively microbiologically proven infection.

Acute GVHD was independently associated with improved overall survival in the landmark analyses, whereas chronic GVHD was not statistically significant after adjustment. One possible interpretation is that this may reflect the graft-versus-leukemia effect observed following allogeneic transplantation consistent with previous reports demonstrating improved disease control among patients who develop acute GVHD [23,24,25,26].

Multivariable analysis also identified salvage treatment intent and disease relapse as independent risk factors for inferior OS following TBI-based allo-HSCT, consistent with previous literature [27,28].

This study has important limitations. It is a retrospective analysis, and pulmonary function testing was not available for all patients. Bronchoscopy and BAL were performed according to clinical indication rather than a standardized protocol, limiting detailed analyses of microbiological findings and preventing systematic comparisons between infectious pulmonary complications and IPS. Furthermore, detailed three-dimensional lung dosimetry was not performed on all patients. Future prospective multicenter studies incorporating standardized diagnostic protocols, comprehensive microbiological investigations, longitudinal pulmonary function assessment, and modern intensity-modulated TBI techniques will be important to further characterize pulmonary complications following HSCT. The extrapolation of survival outcomes is limited by variability in disease biology, treatment response, and patient-specific factors, which may confound direct attribution to pulmonary complications alone.

## 5. Conclusions

In summary, this study provides contemporary data on pulmonary complications following TBI-based myeloablative conditioning using a standardized institutional TBI technique. Consistent with previous literature, idiopathic pneumonia syndrome (IPS) was an uncommon complication. No significant associations were identified between the radiation parameters evaluated in this study and IPS; however, these findings should be interpreted cautiously given the limited number of IPS events and the absence of significant variation in the lung dosimetric data. Utilization of early imaging and bronchoscopy for patients with symptoms of pulmonary toxicity (PT) could be helpful in distinguishing infectious from non-infectious PT. In addition, variables such as age, performance status, comorbidities, pulmonary function tests (PFT) and donor type could be evaluated prospectively in a larger cohort for development of a future risk model. Further prospective studies incorporating intensity-modulated TBI, detailed PFT tracking, and infection risk stratification will be helpful to improve pulmonary outcomes in this population.

## Figures and Tables

**Table 1:** Baseline characteristics for patients undergoing TBI before allo-HSCT from 2015 to 2022.

Characteristic	Number (%) (N = 110)
**Age–median (IQR)**	35.9 (IQR 26.1–44.8)
**ECOG**	
<2	107 (97.3%)
2	3 (2.7%)
**Diagnosis**	
ALL	50 (45.5%)
AML/MDS	24 (21.8%)
ABL/undiff	12 (10.9%)
NHL	19 (17.3%)
CML/myelofibrosis	5 (4.5%)
**Respiratory Comorbidity**	
Yes	21 (19.1%)
No	89 (80.9%)
**Tobacco Use**	
Yes	23 (20.9%)
No	81 (73.6%)
N/A	6 (5.5%)
**HLA Matching**	
Incomplete	78 (70.9%)
Complete	32 (29.1%)
**Intent of Therapy**	
Primary	93 (84.5%)
Salvage	17 (15.5%)
**CNS Involvement**	
Yes	10 (9.1%)
No	100 (90.9%)

**Table 2:** Clinical factors and radiation metrics by PT status (within 100 days).

	No PT (N = 59)	PT (N = 51)	Relevant Test Statistics *	*p*-Value
**Age**			W = 1527	
Median [Q1, Q3]	36.4 [26.6, 44.6]	35.2 [25.5, 45.3]		0.895
**ECOG**			$\chi^{2}\left(1\right)=0.173$	
>0	36 (61.0%)	34 (66.7%)		0.678
0	23 (39.0%)	17 (33.3%)		
**HLA matching**			$\chi^{2}\left(1\right)=0.064$	
Incomplete	15 (25.4%)	15 (29.4%)		0.8
Complete	44 (74.6%)	36 (70.6%)		
**Respiratory comorbidity**			$\chi^{2}\left(1\right)=0.013$	
No	47 (79.7%)	42 (82.4%)		0.908
Yes	12 (20.3%)	9 (17.6%)		
**Tobacco use**			$\chi^{2}\left(1\right)=0.155$	
No	48 (81.4%)	39 (76.5%)		0.694
Yes	11 (18.6%)	12 (23.5%)		
**Prescribed dose**			Fisher’s exact test	
1200 cGy	50 (84.7%)	48 (94.1%)		0.137
1350 cGy	9 (15.3%)	3 (5.9%)		
**Treatment time per field (mins)**			W = 1325.5	
Median [Q1, Q3]	8.88 [7.15, 11.4]	9.25 [7.99, 11.4]		0.284
**TBI Mid-lung Dose Rate (cGy/min)**			W = 1620	
Median [Q1, Q3]	11.1 [8.34, 13.4]	10.6 [8.48, 12.5]		0.49
**Acute GVHD**			$\chi^{2}\left(1\right)=0.041$	
No	23 (39.0%)	18 (35.3%)		0.84
Yes	36 (61.0%)	33 (64.7%)		
**Chronic GVHD**		4	$\chi^{2}\left(1\right)=0.22$	
No	31 (52.5%)	30 (58.8%)		0.639
Yes	28 (47.5%)	21 (41.2%)		

* Relevant test statistics are chi-square values for chi-square tests, Wilcoxon statistics for the Wilcoxon rank-sum test. Fisher’s test gives exact *p*-values. All continuous variables were not normally distributed based on the Shapiro–Wilk test and the non-parametric Wilcoxon rank-sum test were used.

**Table 3:** Clinical factors and radiation metrics by total PT status (within and after 100 days).

	No PT (N = 31)	PT (N = 79)	Relevant Test Statistics *	*p*-Value
**Age**			W = 1420	
Median [Q1, Q3]	38.1 [29.9, 46.9]	34.0 [25.6, 44.1]		0.195
**ECOG**			$\chi^{2}\left(1\right)=$ 0.292	
>0	18 (58.1%)	52 (65.8%)		0.589
0	13 (41.9%)	27 (34.2%)		
**HLA matching**			$\chi^{2}\left(1\right)=0.248$	
Incomplete	10 (32.3%)	20 (25.3%)		0.619
Complete	21 (67.7%)	59 (74.7%)		
**Tobacco use**			$\chi^{2}\left(1\right)=1.106$	
No	22 (71.0%)	65 (82.3%)		0.293
Yes	9 (29.0%)	14 (17.7%)		
**Prescribed dose**			Fisher’s exact test	
1200 cGy	25 (80.6%)	73 (92.4%)		0.0935
1350 cGy	6 (19.4%)	6 (7.6%)		
**Treatment time per field (mins)**			W = 1089.5	
Median [Q1, Q3]	8.73 [7.10, 11.7]	9.17 [7.56, 11.4]		0.371
**TBI Mid-lung Dose Rate (cGy/min)**			W = 1290.5	
Median [Q1, Q3]	11.3 [8.18, 13.3]	10.6 [8.49, 13.0]		0.663
**Acute GVHD**			$\chi^{2}\left(1\right)=0.171$	
No	13 (41.9%)	28 (35.4%)		0.679
Yes	18 (58.1%)	51 (64.6%)		
**Chronic GVHD**			$\chi^{2}\left(1\right)=0.52$	
No	15 (48.4%)	46 (58.2%)		0.471
Yes	16 (51.6%)	33 (41.8%)		

* Relevant test statistics are chi-square values for chi-square tests and Wilcoxon statistics for the Wilcoxon rank-sum test. Fisher’s test gives exact *p*-values. All continuous variables were not normally distributed based on the Shapiro-Wilk test, and the non-parametric Wilcoxon rank-sum test were used.

**Table 4:** Clinical factors and radiation metrics by Early IPS (≤100 days).

	IPS (N = 8)	No IPS (N = 102)	Relevant Test Statistics *	*p*-Value
**Age**			W = 566	
Median [Q1, Q3]	43.0 [36.0, 48.9]	35.0 [25.6, 44.3]		0.0699
**ECOG**			Fisher’s exact test	
>0	7 (87.5%)	63 (61.8%)		0.254
0	1 (12.5%)	39 (38.2%)		
**HLA matching**			Fisher’s exact test	
Incomplete	2 (25.0%)	28 (27.5%)		1
Complete	6 (75.0%)	74 (72.5%)		
**Respiratory comorbidity**			Fisher’s exact test	
No	6 (75.0%)	83 (81.4%)		0.647
Yes	2 (25.0%)	19 (18.6%)		
**Tobacco use**			Fisher’s exact test	
No	7 (87.5%)	80 (78.4%)		1
Yes	1 (12.5%)	22 (21.6%)		
**Prescribed dose**			Fisher’s exact test	
1200 cGy	7 (87.5%)	91 (89.2%)		1
1350 cGy	1 (12.5%)	11 (10.8%)		
**Treatment time per field (mins)**			W = 361	
Median [Q1, Q3]	8.49 [7.94, 9.75]	9.05 [7.48, 11.6]		0.592
**TBI Mid-lung Dose Rate (cGy/min)**			W = 449.5	
Median [Q1, Q3]	11.0 [9.47, 12.6]	10.9 [8.33, 13.1]		0.637
**Acute GVHD**			Fisher’s exact test	
No	3 (37.5%)	38 (37.3%)		1
Yes	5 (62.5%)	64 (62.7%)		
**Chronic GVHD**			Fisher’s exact test	
No	4 (50.0%)	57 (55.9%)		1
Yes	4 (50.0%)	45 (44.1%)		

* Relevant test statistics are chi-square values for chi-square tests and Wilcoxon statistics for Wilcoxon rank-sum test. Fisher’s test gives exact *p*-values. All continuous variables were not normally distributed based on the Shapiro–Wilk test, and the non-parametric Wilcoxon rank-sum test were used.

**Table 5:** Clinical factors and radiation metrics by cumulative IPS.

	IPS (N = 20)	No IPS (N = 90)	Relevant Test Statistics *	*p*-Value
**Age**			W = 1131	
Median [Q1, Q3]	39.7 [35.1, 47.5]	34.0 [25.5, 44.1]		0.074
**ECOG**			$\chi^{2}\left(1\right)=$ 0.83	
>0	15 (75.0%)	55 (61.1%)		0.362
0	5 (25.0%)	35 (38.9%)		
**HLA Matching**			$\chi^{2}\left(1\right)<0.001$	
Incomplete	5 (25.0%)	25 (27.8%)		1
Complete	15 (75.0%)	65 (72.2%)		
**Tobacco Use**			Fisher’s exact test	
No	19 (95.0%)	68 (75.6%)		0.0682
Yes	1 (5.0%)	22 (24.4%)		
**Prescribed dose**			Fisher’s exact test	
1200 cGy	18 (90.0%)	80 (88.9%)		1
1350 cGy	2 (10.0%)	10 (11.1%)		
**Treatment time per field (mins)**			W = 833	
Median [Q1, Q3]	8.98 [7.59, 10.7]	9.03 [7.48, 12.0]		0.606
**TBI Mid-lung Dose Rate (cGy/min)**			W = 976.5	
Median [Q1, Q3]	10.6 [9.08, 12.8]	11.0 [8.24, 13.2]		0.556
**Acute GVHD**			$\chi^{2}\left(1\right)=$ 0.286	
No	9 (45.0%)	32 (35.6%)		0.593
Yes	11 (55.0%)	58 (64.4%)		
**Chronic GVHD**			$\chi^{2}\left(1\right)<0.001$	
No	11 (55.0%)	50 (55.6%)		1
Yes	9 (45.0%)	40 (44.4%)		

* Relevant test statistics are the t-value for *t*-tests, chi-square value for chi-square tests, Wilcoxon statistics for Wilcoxon rank-sum test. Fisher’s test gives exact *p*-values.

**Table 6:** Landmark 100-day univariate analysis for overall survival.

Characteristic	HR *	z-Score	95% CI *	*p*-Value
**Age**	0.97	−1.79	0.94, 1.00	0.074
**ECOG**				
>0	—	—	—	
0	0.79	−0.666	0.39, 1.60	0.5
**HLA Matching**				
Incomplete	—	—	—	
Complete	1.29	0.599	0.56, 2.98	0.5
**Diagnosis**				
ALL	—	—	—	
AML/MDS	0.66	−0.554	0.16, 2.83	0.6
ABL/undiff	2.99	2.19	1.12, 7.94	0.028
NHL	0.87	−0.245	0.30, 2.55	0.8
CML/myelofibrosis	0.00	−0.005	0.00, Inf	>0.9
**Intent of Therapy**				
Primary	—	—	—	
Salvage	2.26	2.08	1.05, 4.86	0.038
**CNS**				
No	—	—	—	
Yes	2.67	2.02	1.03, 6.95	0.043
**Minimal Residual Disease**				
No	—	—	—	
Yes	1.07	0.145	0.41, 2.80	0.9
**Disease Relapse**				
No	—	—	—	
Yes	7.01	4.96	3.25, 15.1	<0.001
**Acute GVHD**				
No	—	—	—	
Yes	0.46	−2.21	0.23, 0.92	0.027
**Chronic GVHD**				
No	—	—	—	
Yes	0.59	−1.50	0.29, 1.18	0.13
**IPS within 100 days**				
IPS	—	—	—	
no IPS	1.25	0.310	0.30, 5.25	0.8
**PI within 100 days**				
no PI	—	—	—	
PI	3.02	3.09	1.50, 6.09	0.002

* Abbreviations: CI = Confidence Interval, HR = Hazard Ratio.

**Table 7:** Landmark 100-day multivariate analysis for overall survival.

Characteristic	HR *	95% CI *	*p*-Value
**Age**	0.98	0.94, 1.02	0.4
**Diagnosis**			
ALL	—	—	
AML/MDS	1.02	0.20, 5.34	>0.9
ABL/undiff	2.40	0.83, 6.94	0.11
NHL	1.38	0.43, 4.39	0.6
CML/myelofibrosis	0.00	0.00, Inf	>0.9
**Intent of Therapy**			
Primary	—	—	
Salvage	2.70	0.87, 8.35	0.085
**CNS**			
No	—	—	
Yes	2.24	0.59, 8.46	0.2
**Disease Relapse**			
No	—	—	
Yes	3.24	1.35, 7.74	0.008
**Acute GVHD**			
No	—	—	
Yes	0.38	0.17, 0.85	0.017
**Chronic GVHD**			
No	—	—	
Yes	0.52	0.23, 1.21	0.13
**PI within 100 days**			
no PI	—	—	
PI	2.38	1.01, 5.62	0.047

* Abbreviations: CI = Confidence Interval, HR = Hazard Ratio.

**Table 8:** Landmark 100-day multivariate analysis–final stepwise model.

Characteristic	HR *	95% CI *	*p*-Value
**Intent of Therapy**			
Primary	—	—	
Salvage	2.92	1.09, 7.84	0.034
**CNS**			
No	—	—	
Yes	2.72	0.76, 9.74	0.12
**Disease Relapse**			
No	—	—	
Yes	3.69	1.58, 8.62	0.003
**Acute GVHD**			
No	—	—	
Yes	0.44	0.21, 0.95	0.036
**Chronic GVHD**			
No	—	—	
Yes	0.52	0.24, 1.14	0.10
**PI within 100 days**			
no PI	—	—	
PI	2.57	1.13, 5.84	0.024

* Abbreviations: CI = Confidence Interval, HR = Hazard Ratio.

## Data Availability

Research data are stored in an institutional repository and will be shared upon request to the corresponding author.

## Supplementary Materials

The following supporting information can be downloaded at: https://www.mdpi.com/article/10.3390/curroncol33090545/s1, Figure S1: Monte Carlo simulated lung DVH data for 10 patients receiving 1200 cGy prescribed dose; Table S1: Mean lung dose and V 1200 cGy lung.

## Abbreviations

AFT	Accelerated Failure Time
ALL	Acute Lymphoblastic Leukemia
AML	Acute Myeloid Leukemia
ATS	American Thoracic Society
AIC	Akaike Information Criterion
BAL	Bronchoalveolar Lavage
COG	Children’s Oncology Group
CT	Computed Tomography
CXR	Chest X-ray
ECOG	Eastern Cooperative Oncology Group
GVHD	Graft-versus-Host Disease
HSCT	Hematopoietic Stem Cell Transplantation
IPS	Idiopathic Pneumonia Syndrome
MDS	Myelodysplastic Syndrome
MRD	Minimal Residual Disease
OS	Overall Survival
PFT	Pulmonary Function Test
PI	Pulmonary Infection
PT	Pulmonary Toxicity
RCT	Randomized Controlled Trial
TBI	Total Body Irradiation
VMAT	Volumetric Modulated Arc Therapy
