# Supplementary material for: Idiopathic Pneumonia Syndrome and Pulmonary Toxicity After Total Body Irradiation for Bone Marrow Transplantation in the Modern Transplant Era
Source: Curr Oncol. 2026 Sep 9;33(9):545. doi: 10.3390/curroncol33090545 (PMC13605090; doi:10.3390/curroncol33090545)
Supplement: Supplementary file 1 [file curroncol-33-00545-s001.zip › curroncol-4557285-supplementary.pdf]

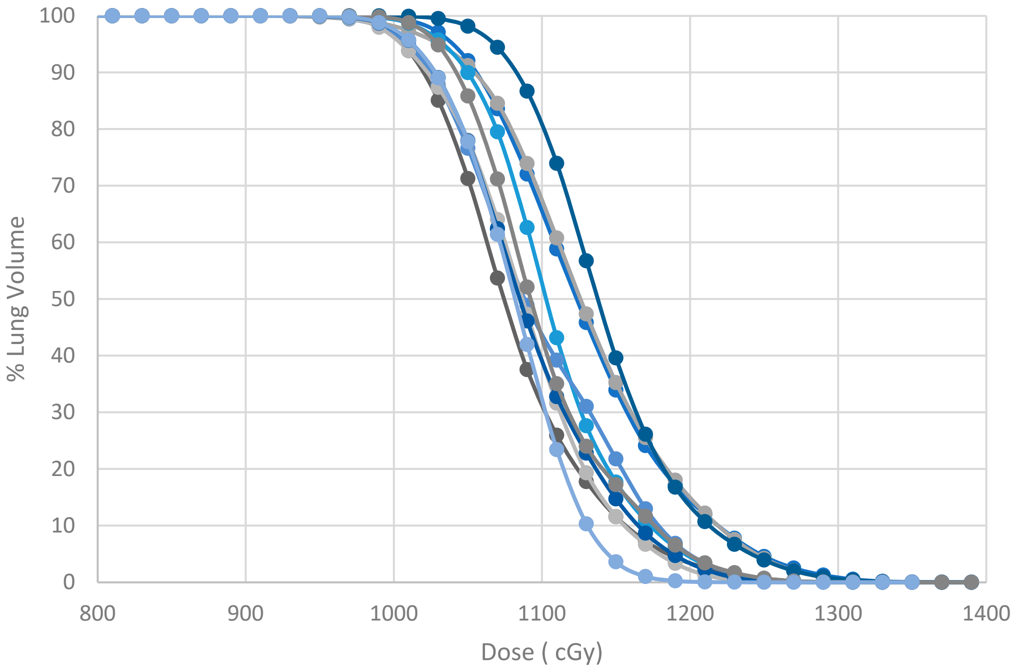

Figure S1. Monte Carlo Simulated Lung DVH data for 10 patients receiving 1200 cGy prescribed dose.

Table S1. Mean lung dose and V 1200 cGy lung.

	**Min**	**Max**	**Median**
Mean Lung Dose (cGy)	1050	1140	1090
V 1200cGy Lung	0.14%	15.1%	5.04%
